# Exploring recruitment, willingness to participate, and retention of low-SES women in stress and depression prevention

**DOI:** 10.1186/1471-2458-10-588

**Published:** 2010-10-05

**Authors:** Judith EB van der Waerden, Cees Hoefnagels, Maria WJ Jansen, Clemens MH Hosman

**Affiliations:** 1 Department of Health Promotion, Maastricht University, Maastricht, the Netherlands; 2 Trimbos Institute, Netherlands institute for mental health and addiction, Utrecht, the Netherlands; 3 Public Health Services South Limburg, Geleen, the Netherlands; 4 Behavioural Science Institute, Radboud University Nijmegen, Nijmegen, the Netherlands; 5 CAPHRI School for Public Health and Primary Care, Maastricht University, Maasrticht, the Netherlands

## Abstract

**Background:**

Recruitment, willingness to participate, and retention in interventions are indispensable for successful prevention. This study investigated the effectiveness of different strategies for recruiting and retaining low-SES women in depression prevention, and explored which sociodemographic characteristics and risk status factors within this specific target group are associated with successful recruitment and retention.

**Methods:**

The process of recruitment, willingness to participate, and retention was structurally mapped and explored. Differences between women who dropped out and those who adhered to the subsequent stages of the recruitment and retention process were investigated. The potential of several referral strategies was also studied, with specific attention paid to the use of GP databases.

**Results:**

As part of the recruitment process, 12.1% of the target population completed a telephone screening. The most successful referral strategy was the use of patient databases from GPs working in disadvantaged neighborhoods. Older age and more severe complaints were particularly associated with greater willingness to participate and with retention.

**Conclusions:**

Low-SES women can be recruited and retained in public health interventions through tailored strategies. The integration of mental health screening within primary care might help to embed preventive interventions in low-SES communities.

## Background

Women with low socioeconomic status (SES) and those living in disadvantaged circumstances are at high risk for not only physical health problems, but also mental health problems like depression [1,2]. Although in recent years effective interventions have been developed for primary prevention of depression, low-SES women who are at high risk are often difficult to reach with preventive mental health services [3,4]. Further, they drop out of preventive interventions more frequently than their wealthier or more highly educated counterparts [5], resulting in low retention rates [6,7]. Recruitment, willingness to participate, and retention in interventions are indispensable for successfully reducing public health problems such as depression [8]. However, knowledge on which recruitment techniques are most effective for this population, or which characteristics of low-SES women are associated with their successful participation and retention in interventions is limited.

The most common methods for intervention recruitment are self-referral in response to announcements in local newspapers, TV and radio programs, and mass mailings [9,10]. However, previous research has indicated that a more personalized approach might be more suitable for recruiting disadvantaged women and educating them about the availability and utility of mental health services [11,12]. This approach might entail phone calls, targeted mailings, face-to-face referrals, and consultations by local community services [13-15]. Simultaneous use of multiple methods allows mental healthcare providers to reach a diverse ethnic and socioeconomic sample [10], but also implies the need for a more active role in contacting potential participants. To increase disadvantaged women's willingness to participate, intervention strategies should be adapted to their needs and values [16-18]. With respect to retention, several barriers and facilitators have been found to affect low-SES women's use of preventive mental health services. Instrumental barriers to retention relate to costs, transportation, and time [6,13,19], while psychological barriers are associated with attitudes and beliefs about mental health and concerns about stigma [13,17]. Removing barriers, for instance through increased flexibility of services, generally increases service adherence [14,17,20].

As in several industrialized nations, the problem of recruitment and retention of disadvantaged populations in prevention is also pertinent in the Netherlands. No more than about 4000 people are reached annually with all activities for indicated depression prevention. This constitutes approximately 1% of the 359,000 people who develop depression each year, and an even lower percentage of those at risk for depression [21]. Moreover, participants in preventive mental health services generally have higher education levels, suggesting an underrepresentation of people with a lower socioeconomic or migrant status [22].

To increase participation by this group, it is necessary to improve our knowledge on factors affecting their recruitment, willingness to participate, and retention in depression prevention. This study seeks to structurally map and explore this process for a preventive depression course targeted at women from disadvantaged communities.

The first aim of this study was thus to explore the effectiveness of different strategies for recruiting low-SES women from disadvantaged communities in this preventive intervention. A second aim was to identify which sociodemographic characteristics and risk status factors *within *the specific target group of low-SES women affect their successful recruitment and retention. A final aim was to determine our overall success rate in reaching our target population. Findings from this study could help providers understand how to better engage low-SES women in public mental health and which women from this high-risk group are more likely to participate in interventions aimed at preventing depression.

## Methods

### Sample

The target group for the present study was low-SES women aged 20-55 years, with elevated stress or depressive symptoms. They were recruited for participation in the Exercise without Worries (EWW) prevention course. EWW is provided by the prevention department of the district community mental health center in collaboration with the local Public Health Service. The main goal of this intervention is to reduce stress and depressive complaints and increase coping related competences by empowering the women through their strengths and resilience. In eight two-hour sessions physical exercise and psycho-education are combined to address evidence-based psychosocial risk factors for stress and depression in low-SES women. Topics covered in the psycho-education relate to specific problems associated with low-SES status and deal with issues such as depression, recognition of signals of tension in the body, constructive thinking and assertiveness. The exercise component focuses on stretching, muscle reinforcement, flexibility, body focused exercise and relaxation. The core element of the EWW course is its group-based format in which psycho-educative topics are combined with body-focused exercises. In each session, psycho-education and exercise components are coordinated as far as possible in an effort to reciprocally reinforce the message. The intervention features are described in more detail elsewhere [23].

Recruitment took place in a southern Dutch city with 36 residential neighborhoods, nine of which are considered to be disadvantaged [24]. The city is fairly homogeneous in ethnic terms and counts 120,175 inhabitants, 31,657 of whom are women aged 20-55 [25]. Since years of formal education is found to be a valid single estimator of socioeconomic status [26], we used this to determine the size of the adult female low-SES population. Of the city population, 34% has completed 10 years or less of formal education, leading to an estimation of approximately 10,763 adult women as low-SES. To determine our overall success rate in reaching our target population, we used the same approach to calculate a theoretical maximum target population in the nine disadvantaged neighborhoods. The population of women aged 20-55 in these nine neighborhoods was estimated at 12,029, of whom 4289 had 10 years or less of formal education [24]. A recent health survey by the district Public Health Service showed that 53.5% of low-SES women had experienced moderate to severe depressive symptoms in the past week [27]. This would imply that 2295 (53.5% of 4289) women living in those neighborhoods were estimated to have moderate to severe depressive symptoms.

### Procedures

**Figure 1:**
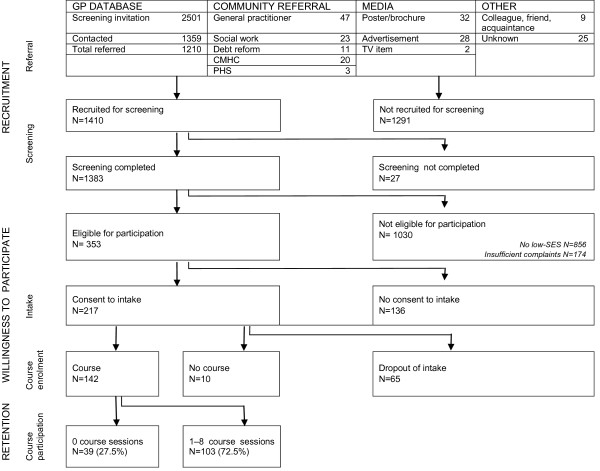
**Schematic overview of the recruitment and retention process**.

Figure 1 systematically describes the recruitment and retention process, which consists of the following steps: *recruitment *(referral and screening), *willingness to participate *(intake and course enrolment), and *retention *(course participation). Recruitment took place between April 2005 and November 2007, involving several referral strategies to engage low-SES women. Twenty general practitioners (GPs) working in or near the targeted socioeconomically deprived neighborhoods were invited to identify from their caseloads women who might be eligible for participation, and referring them to our team for a short telephone screening. Five of these GPs gave us permission to approach all adult women (20-55 years) in their patient databases. These women were sent a letter explaining the aim of the telephone screening and asking for their participation. They were then contacted by phone one week later. Other strategies focused on enhancing direct referrals by providing information about EWW to social work and debt repayment services, and to the district mental health centre and Public Health Service. Women were also referred through posters and brochures in locations frequented by low-SES women. Finally, advertisements were placed in local newspapers, neighborhood bulletins and on a local television network, and word of mouth advertising was used as well. Including these strategies lead to inevitable cross-over of information about the intervention to other parts of the city as well.

All women referred to EWW through one of these methods were screened for stress and depressive complaints in a 10-minute telephone interview conducted by trained lay interviewers. During the screening, the women provided demographic information about their age, nationality, marital status, number of children living at home, educational attainment, occupational situation, and monthly family income. They completed the 10-item version of the Perceived Stress Scale (PSS) [28] to assess the frequency of stress-inducing situations and feelings of stress over the past month. They also completed the 10-item version of the Center for Epidemiological Studies Depression (CES-D) [29] scale to determine the presence of depressive symptoms in the past week.

The next recruitment phase consisted of inviting those women who fulfilled low-SES criteria and stress or depression scores above the cut-off levels to participate in the EWW course. Those who had elevated symptom scores but did not satisfy the demographic criteria were referred to regular mental healthcare. Women who met the participation criteria and were willing to participate were scheduled for an intake meeting with an intervention staff member. Women who did not keep this appointment were contacted to schedule a new meeting, with a maximum of five attempts. The intake served multiple purposes: firstly, to gather relevant background information on the women's psychological complaints, physical restraints that might hinder their participation, and need for clinical treatment; secondly, to explain the course objectives and respond to questions; and thirdly, to establish contact between the women and the intervention provider. The intakes were preferably conducted at the local women's health center to avoid any stigma associated with mental health centers. During the course, attendance was registered and women who missed a meeting without having notified the staff in advance were contacted about their absence. Approval for conducting this study was provided by the Medical Ethics Committee of the Academic Hospital Maastricht/Maastricht University, the Netherlands, reference number MEC 05-004.

### Data analysis

Differences in sociodemographic characteristics were compared between women who completed the telephone screening and those who dropped out during recruitment. Chi-square analyses were applied for categorical variables, t-tests, and analyses of variance for continuous variables respectively. Bonferroni corrections were applied for multiple comparisons within each separate phase. The variables were age, nationality, educational level, marital status, employment status, neighborhood prosperity score, and PSS and CESD scores. The type of recruitment strategy (i.e., GP database, community referral, media, or other) was tested as well, to examine whether it was related to successful completion of the screening. The same analyses were used to investigate sociodemographic characteristics and risk status factors associated with participation and successful retention in the course. To determine our overall success rate in reaching our target population, we compared a subsample of the women reached with the EWW intervention and residing in nine disadvantaged neighborhoods with the theoretical maximum target population living in those neighborhoods. We then estimated again the outcomes for recruitment, willingness to participate, and retention for our subsample, to compare with this theoretical maximum.

## Results

### Recruitment

A screening invitation was sent to 2501 women who were identified in the GP databases. We were able to contact 1359 of these women (54.3%), 149 of whom refused participation. This resulted in the recruitment of 1210 women (48.4% of those who were sent an invitation) for the screening interview through the GP databases. For the other recruitment strategies, none of the women refused to participate in the screening, resulting in 104 women recruited through community referral, 62 through the media, and 34 by other means. Of the 1410 women who were reached for the telephone screening, 1383 (98.1%) completed the interview (see table 1).

**Table 1:** Success of different recruitment strategies compared to retention rate for completion of screening

Recruitment strategy	Referred for screening N	Screening completed N	Eligible N	Consent to intake N	Participation in course N
GP database	2501	1194	256	124	76
Community referral	104	96	58	55	38
Media	62	60	22	21	14
Other	34	33	17	17	14
Total	2701	1383	353	217	142

Of the total number of women screened, 856 (61.8%) did not meet low-SES criteria. Of those who did, 174 (12.6%) scored below the cut-off levels for stress and/or depressive symptoms. This left 353 women who were eligible for participation in the course.

### Willingness to participate

Appointments for the intake were made for 217 (61.2%) of the eligible women. Women with complaints above the cut-off levels who did not consent to an intake had lower stress (*t *(345) = - 4.33, *p *< .000) and depressive symptom (*t *(344) = -5.12, *p *< .000) scores at screening than women who consented. Most women attended the first appointment. In 32.7% of the cases where the women did not attend the first appointment, two or more additional appointments had to be scheduled before the intake took place. Overall, 152 (70.0%) women completed the intake. Reasons for not participating in the intake were: lost interest (66.1%), no reason given (18.5%), no time (10.8%), course no longer needed (1.5%), and medical illness (3.1%). Those women who did not attend the intake were more often younger (*M *= 42.55, *SD *= 10.26 vs. *M *= 45.26, *SD *= 9.44; *p *= .007) and had lower depressive symptom scores (*M *= 14.92, *SD *= 7.24 vs. *M *= 16.75, *SD *= 6.58; *p *= .006). Whether or not the intake was completed was unrelated to the recruitment strategy that was used.

### Retention

Of the 152 women who completed the intake, 2.0% were excluded from participation in the course due to overly severe psychological or medical problems. A further 2.4% lost interest after receiving additional information during the intake, and 2.2% were unavailable on the days/at the times the course was offered, thus leaving 142 participants. Between September 2005 and May 2008, a total of 30 EWW groups started with an average of 9.6 participants (*SD *= 1.96, range 5-13) per group. Almost 30% of the eligible participants who consented to take part in the course failed to attend. For the 103 participants, attendance ranged from one to eight meetings, with a mean of 5.62 (*SD *= 2.16); 18 women (12.7%) completed all eight meetings. Overall, the only characteristic associated with course completion was age. Women who completed the course were older than those who did not attend all meetings (*p *< .001). Other demographic variables and risk status were unrelated to course attendance, as was recruitment strategy (*p *> .01). The outcomes on stress and depressive symptoms for the participants that were successfully recruited and retained in the intervention will be tested separately in an effect evaluation.

### Reach of target population in low-SES neighborhoods

**Figure 2:**
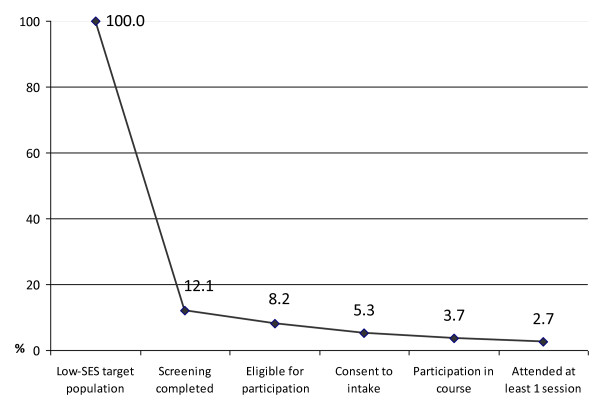
**Reach of intended target population (N = 2295) per recruitment phase**.

The data reported above concern the total recruited sample originating from all neighborhoods. To determine our overall success rate in reaching our target population, we compared a subsample of women living in nine disadvantaged neighborhoods to the theoretical maximum of 2295 low-SES women with depressive complaints living in these neighborhoods. From this selected population we were able to contact 277 women (12.1%) for the screening interview. As table 2 shows, 189 women were eligible for participation in the course. Of the 121 women who consented to an intake, 90 (74.4%) completed the intake and 84 were willing to participate in the course. This means that 3.7% of the 2295 women were reached and that 2.7% attended the course (figure 2).

**Table 2:** Reach of intended target population (N = 2295) per recruitment phase

Phases		N	% in population	% in recruited population	% in eligible population
***Theoretical maximum population***	Low-SES women with depressive symptoms	2295	100.0	-	-
***Recruitment***	Screening completed	277	12.1	100.0	-
	Eligible for participation	189	8.2	68.2	100.0
***Willingness to participate***	Consent to intake	121	5.3	43.7	64.0
	Participation in course	84	3.7	30.3	44.4
***Retention***	Attended at least 1 session	61	2.7	22.0	32.3

## Discussion

Effective recruitment, willingness to participate, and retention of low-SES women in preventive interventions is critical for reducing depression in this group. To this end, we structurally mapped and explored this process for a preventive depression course targeted at women from disadvantaged communities. Based on the most conservative rate, at least 12.1% of the target population in nine specific neighborhoods completed a telephone screening as part of a targeted recruitment process. Most women were referred through the patient databases of GPs working in disadvantaged neighborhoods. Compared to strategies like community or media referral, this method resulted in more women being recruited for initial contact. Particularly older women and those with more severe complaints at screening were willing to participate and were retained in the intervention.

The effectiveness of the GP database as a referral strategy was most visible in the first stage of the recruitment process, in that it led to the largest number of initial referrals to the course. After this phase all four recruitment strategies showed equal success in terms of willingness to participate and retention in the intervention. Other effective ways to engage women were outreach through local community services and referral persons, a finding that corroborates previous research [10,12]. Further, we found that the use of carefully selected media is a suitable method to reach these women and can support the direct referral by community workers.

Thirty-nine percent of eligible women did not consent to an intake meeting even though their scores indicated an at-risk status. These women may have felt that their functioning and mental health were not heavily affected, and thus that they did not particularly need to participate in a prevention program [30]. We tried to increase willingness to participate by removing instrumental barriers. Despite the fact that financial barriers are at most a minor impediment for disadvantaged populations to access mental healthcare in the Netherlands [31], the EWW course was offered free of charge, and additional expenses for childcare and public transport were reimbursed. Also, the intervention was presented and executed as a course rather than as therapy, aiming to avoid stigmatization. Still, among those women who consented to an intake, 30.0% did not actually attend it. One explanation might be that low-SES women are often uncomfortable saying no and will thus passively assent to appointments [12], resulting in non-attendance.

Finally, increasing retention during the intervention appears to be especially important. Almost 30% of the women who consented to participate in the course did not attend at all, and only 12.7% completed all sessions. The low retention might influence the overall outcomes of the EWW intervention and will be considered as a potential influential factor in the effects analyses. Insufficient participation may decrease not only the intervention's effect for the women involved [32], but also the cohesion and trust within the group, and therefore potentially affect all group members. Dropouts were often younger, which corroborates the findings from previous research [32]. To facilitate attendance, participants were called and reminded of the course shortly before its start, and those who missed a session without giving prior notification were also contacted. However, we were unable to determine whether these measures resulted in increased attendance. Other studies have shown that telephone prompting, letters, and consultations are effective means of increasing attendance rates [13,14].

In this study we were able to reach around 12% of the target population of low-SES women with a telephone screening, and 3.7% ultimately participated in the intervention. Of the people who develop depression each year in the Netherlands, only about 1% participates in preventive interventions. Moreover, these participants often belong to higher socio-economic strata, suggesting that participation might possibly be even lower for low-SES groups [21,22]. In this study the reach was almost four times higher than the national level and possibly even higher considering that it was achieved among a low-SES population known to be hard to reach with mental health services.

One limitation of this study is that we used years of formal education as a single estimate to determine the socio-economic status of our study population. Socioeconomic status is a complex concept that is assessed using a variety of different measures. These include income, material possessions, occupational status and education, which are the concepts most commonly studied. Years of formal education has been shown to be a very good indicator of long-term economic position since it often precedes and influences employment, work, earnings, and income, thus acting as a key to positions in the stratification system [33,34]. Furthermore, it appears that educational level is the socioeconomic indicator that is most strongly linked to mental well being and common mental disorders [35], especially so for people with few alternative resources, such as disadvantaged women [33,34]. Nevertheless, it is possible that by using this measure we might have excluded those women who have a high educational level, but who are unemployed or have low monthly incomes. Minority immigrants in particular may be disadvantaged in income attainment, as they often have access only to those sectors with lower earning potential [36,37]. However, the ethnic composition of the study location, in which 20% of the population are of non-Dutch nationality, and only 6% of non-Western origin [25], makes it less likely that our sample has been strongly biased in this respect.

Another limitation of this study is that contacting women for a screening via telephone means that only those with a valid number can be reached. Further, we aimed to recruit women from specific socioeconomically deprived neighborhoods, yet low-SES communities generally have high residential mobility. Thus, numerous addresses in the GP databases were not current, which may have prevented us from reaching some of our target group. Finally, we only offered one intervention. Some women may have been less willing to participate because this particular intervention did not appeal to them, or that more might have participated if we had been able to propose a multicomponent approach.

Despite the importance of successfully recruiting and retaining low-SES women in public mental health interventions, this study showed that women with stress or depressive symptoms were infrequently referred by community-based services providers. Although successful programs for depression prevention and treatment are available [38,39], professionals need to become more aware that disadvantaged populations are less likely to participate in such interventions. Screening women at high risk for increased depressive or stress symptoms should become a standard procedure for those providers who have frequent contact with women from disadvantaged communities. A workable solution might be 'stepped' referral, in which primary care practitioners or mental health professionals form trusted sources in the community who can identify high-risk women and subsequently refer them for screening. However, more fine-tuning is needed to identify which people within the high-risk groups need to be targeted for screening efforts. Overall, it is important that screening efforts are embedded in an appropriate care structure, so that women are not only screened for the presence of mental health problems, but also receive suitable subsequent care [40-42]. Particularly if a large range of preventive interventions are offered for women to choose from, their willingness to participate might increase.

## Conclusions

Continued systematic investigation into recruitment and retention of low-SES groups has the potential to contribute significantly to the science of prevention. Although low-SES women are underrepresented in public health interventions, this study showed that it is possible to recruit and retain such women in a preventive intervention to a larger extent than even the middle class in the Netherlands. Integrating active recruitment and screening methods with ongoing primary care might help to embed preventive services within the settings and communities in which these women live and create win-win combinations of disadvantaged women and health care professionals.

## Abbreviations

CES-D: Center for Epidemiological Studies Depression scale; CMHS: Community Mental Health Center; EWW: Exercise Without Worries; GP: General Practitioner; PHS: Public Health Service; PSS: Perceived Stress Scale; SES: Socioeconomic Status

## Competing interests

The authors declare that they have no competing interests.

## Authors' contributions

JW contributed to the design and conduct of the study, monitored recruitment and data collection, performed substantial statistical analyses, and helped interpret the results and draft and review the manuscript. CH participated in the study design, data analysis, interpretation of results, and drafting and review of the manuscript. MJ contributed to the conception and design of the study and helped critically revise the manuscript in terms of content. CMHH contributed to reviewing and improving the manuscript. All authors read and approved the final manuscript.

## Pre-publication history

The pre-publication history for this paper can be accessed here:

http://www.biomedcentral.com/1471-2458/10/588/prepub
